# On the Comparison of Wearable Sensor Data Fusion to a Single Sensor Machine Learning Technique in Fall Detection

**DOI:** 10.3390/s18020592

**Published:** 2018-02-14

**Authors:** Panagiotis Tsinganos, Athanassios Skodras

**Affiliations:** Department of Electrical and Computer Engineering, University of Patras, 265 04 Patras, Greece; skodras@ece.upatras.gr

**Keywords:** fall detection, data fusion, accelerometer, gyroscope, smartphone, wearable sensors, mHealth

## Abstract

In the context of the ageing global population, researchers and scientists have tried to find solutions to many challenges faced by older people. Falls, the leading cause of injury among elderly, are usually severe enough to require immediate medical attention; thus, their detection is of primary importance. To this effect, many fall detection systems that utilize wearable and ambient sensors have been proposed. In this study, we compare three newly proposed data fusion schemes that have been applied in human activity recognition and fall detection. Furthermore, these algorithms are compared to our recent work regarding fall detection in which only one type of sensor is used. The results show that fusion algorithms differ in their performance, whereas a machine learning strategy should be preferred. In conclusion, the methods presented and the comparison of their performance provide useful insights into the problem of fall detection.

## 1. Introduction

It is an indisputable fact that the global population is steadily ageing, which presents local communities with new challenges. Governments adopt policies that help individuals live longer and healthier. However, not everyone can reap the benefits of such strategies. Many are afflicted by health conditions and medical problems, such as falls.

According to World Health Organization (WHO), “a fall is defined as an event which results in a person coming to rest inadvertently on a lower level” [1]. Falls may be caused by chronic diseases (arthritis) and visual impairment or hazards in the living environment and dangerous activities. Although injuries from falls are non-fatal, many fall-related deaths happen every year. In addition to physical injuries, falls are liable for psychological conditions, depression, and avoidance of the activity that caused the fall. Finally, they can induce huge healthcare costs, since a fall is regularly followed by recurrent fall incidents that increase hospital admissions and recovery treatments.

Considering how severe the consequences of incurred falls are, it is crucial that fall detection and prevention systems be developed. Should a fall be detected soon enough, then most effective treatments and appropriate interventions can be applied, while medical expenditures are reduced and lives saved. Additionally, if fall prediction is feasible, a protective system can be actuated in order to prevent a fall from happening. A fall detection system has two major functional components: the detection component, and the communication component. The former is responsible for collecting and analyzing sensor data, while the latter notifies relatives and caregivers of a fall. The aim of such a system is to distinguish between a fall and an activity of daily living (ADL).

Advances in sensor technology and signal processing have paved the way for advanced autonomous activity monitoring and fall detection systems. Sensor data are collected and then processed by a signal processing unit to infer information about the posture of the senior. These systems comprise different types of sensors that can be either wearable (e.g., IMU devices, smartphones, smartwatches) or ambient (e.g., PIR, microphones, cameras). In this paper, we concentrate mostly on the use of IMU sensors that constitute custom devices or are embedded in smartphones. These sensors belong to the category of Microelectromechanical sensors (MEMS) that are made on a tiny scale, and their design incorporates a part that physically moves or vibrates. In accelerometers, for example, the deviation of this moving part from the neutral position is analogous to the applied acceleration. There are many configurations for the dynamic range, resolution, and sampling rate characteristics. Typically, for smartphone sensors the dynamic range is 4 g and 0.6 rad/s, the resolution is around 0.1 m/s^2^ and 2 × 10^−5^ rad/s, for accelerometers and gyroscopes, respectively, while the maximum sampling rate is 100 Hz for both types of sensors [2].

Another aspect of a fall detection system is what method is used to detect a fall: Is it a threshold-based algorithm (TBA), or does it utilize machine learning (ML) techniques? In the former, sensor data are constantly monitored, and when the signal exceeds a predetermined value a fall is detected, whereas in the latter signal characteristics extracted from segments of the signal feed a classifier that distinguishes between fall and normal activity.

Although a single sensor may be adequate to distinguish falls from other simple activities, a fusion system can be used for more robust measurements and accurate detection. Data fusion is the process of combining multiple data sources to produce more accurate and useful information. There are multiple ways of developing a data fusion approach. For example, one type of sensor can be positioned in different locations on the body (e.g., an accelerometer attached to the chest, wrist, and hip) or the environment (e.g., vibration sensors on the floor). Alternatively, a multimodal (e.g., comprised of an accelerometer, gyroscope, magnetometer) device can be attached on a single anatomical landmark on the human body (e.g., hip, chest) to offer complementary information about the activity performed. Lastly, the aforementioned method can be enhanced if such sensing devices are placed on different body locations.

Generally, the term sensor fusion refers to techniques that enable the synergistic combination of more than one sensors to get improved performance regarding accuracy and reliability. The employed sensors may be of the same or a different type. In the first case, the acquired information involves redundant measurements that can be used to reduce any inconsistencies—for example, averaging makes the data less noisy—or provide robustness in the case of sensor failure. On the other hand, different types of sensors are used to capture complementary information in order to get the complete picture of the phenomenon or provide information that is not obtainable by any single sensor.

**Figure 1:**
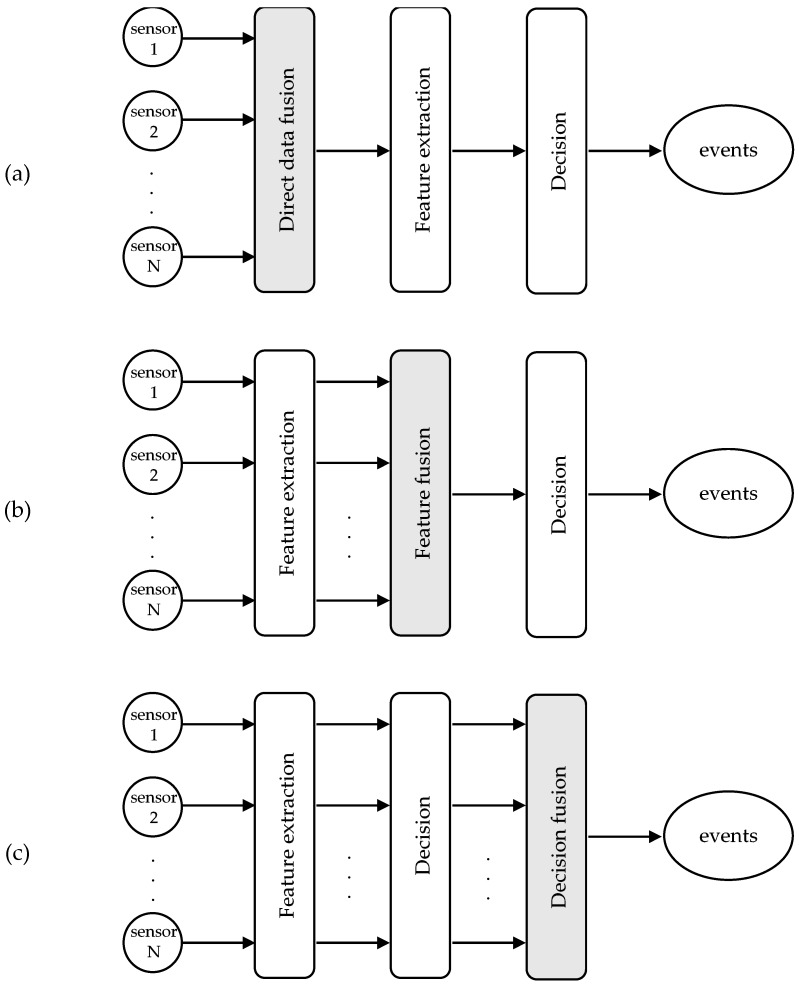
Schematic diagrams showing fusion architectures at data (**a**), feature (**b**), and decision (**c**) levels.

From a data processing perspective, sensor fusion techniques are usually grouped into three levels (Figure 1): direct data fusion, feature fusion, and decision fusion [3]. In case of redundant sensors, the sensor data can be combined directly. Applications of these schemes can be found in optimal averaging of sensor arrays, which aim to adjust the scale and bias coefficients so that the signal-to-noise ratio (SNR) is maximized, and in blind source separation (BSS) problems, where the original signals are extracted from sensors that measure a different combination of the source signals.

The feature-level fusion integrates a set of features extracted from the sensors. These features correspond to the main characteristics of data, while they ignore irrelevant information. Typically, they contain time-domain (signal amplitude, zero-crossings, mean, standard deviation, higher moments), frequency-domain (energy bins, Fourier coefficients, frequency peaks, power spectral density), or time-frequency components (wavelet coefficients). Having extracted a set of features, the role of the feature-level fusion is to select the appropriate features, and in some cases, transform them in a higher or lower dimensionality space, so that the classifier can discriminate the patterns belonging to different classes.

Finally, in the decision level fusion, the decisions of multiple sensors are taken into account to improve the event detection accuracy. The results of the sensors can be merged at various levels, creating a fusion network topology. In large-scale sensor network systems, it may be more advantageous to fuse a few sensors at a local node before transmitting the information over a long distance to the final fusion processor [4]. Common techniques in this type of fusion are classical inference, Bayesian inference, Dempster-Shafer’s theory, and fuzzy logic. Classical inference methods compute an estimate of the joint probability and can test two hypotheses with maximum likelihood decision rules. On the other hand, Bayesian inference incorporates a priori knowledge and new observations about the probability of a hypothesis. The Dempster-Shafer’s method is a generalization of Bayesian inference that assigns a level of uncertainty to the likelihood of the hypotheses. Another set of decision-level fusion techniques is based on fuzzy logic, which combines membership functions and IF-THEN rules.

In the present work, our single sensor fall detection system is compared to three recently proposed IMU sensor-type data fusion schemes. The comparisons are based on three publicly available datasets. It is proven that the performance of the fusion schemes varies with the dataset, while our ML classifier presents improved detection capabilities. The paper is organized as follows. Section 2 examines past and recent advances in the literature of data fusion in activity recognition and fall detection. Section 3 presents the implementation of three fall detection systems that involve the fusion of accelerometer and gyroscope data. In Section 4 and Section 5, the experimentation procedure and the results of the comparison of the methods are presented. Finally, a brief discussion and conclusions are reported in the last section.

## 2. Related Work

Both academia and industry have been interested in fall detection over the last decades [5,6]. There has been developed an array of fall detection systems (Table 1) with varying degrees of accuracy. An extended review of fall detection systems that focus on the use of multisensor fusion-based methods is presented in [6].

When addressing the problem of fall detection, one should decide on what sensors to use, their location, and how to combine the collected data. Many researchers have studied wearable IMU sensors that may be accompanied by cameras or microphones [33], while works in activity recognition usually employ ambient sensors [7,8]. There is also a considerable amount of literature on determining what the best location to attach an accelerometer or gyroscope is [9,10,11,12]. All suggested that the waist-attached sensors should be preferred, whereas head and chest can be used alternatively. The simplest data fusion algorithm is implemented by Casilari et al. ([13]). In their work, they compare 4 threshold-based algorithms (TBAs) that use accelerometer data from a smartphone and a smartwatch. The simplicity of the method is due to the fact that a fall is detected only when the acceleration magnitude in both devices exceeds a threshold within a time interval of 1 s. Their results in terms of sensitivity and specificity (sensitivity = 90%, specificity = 91.7%) can be considered mediocre, since arm movements may be unassociated with the measured activity [12], therefore reducing the performance.

In recent years, there has been a growing interest in combining data from multiple sensors. Great attention has been given to accelerometer and gyroscope sensors, since they are widely available through smartphones. Yang [14] developed a fall detection system based on a TBA that used the acceleration vector magnitude (AVM) along with pitch and roll angular velocities from a smartphone attached to the chest. Similarly, the authors of [15] applied thresholds on acceleration and angular velocity from a custom device to infer whether a fall happened. Other attempts that place the sensing device on the upper trunk include [16,17,18]. In their analysis, Huynh and co-workers [17] improved upon the previous studies with the use of upper and lower thresholds. Analogous results were achieved by authors of [16,18], who developed a k-Nearest Neighbors (kNN) classifier. Features from a sliding window are sent to the classifier, resulting in sensitivity and specificity greater than 95%. In addition, authors of [19] analyze accelerometer and gyroscope data using sliding windows. They propose that sudden changes in the user’s orientation in combination with abrupt variations of the AVM can be a clear sign that a fall occurred. An innovative approach is made by Ando et al. [20]. In their work, they suggest using activity patterns and the correlation function to categorize ADLs and falls. Particularly, the correlation of signal signatures and acceleration segments is compared with thresholds (different for each activity), while angular velocity is used to refine the detection. Apart from accelerometer and gyroscope, other sensors have been used. A barometer and a magnetometer were employed by [21] to help with the detection of fall phases. A quaternion filter extracts acceleration relative to Earth’s frame from the IMU sensors, whereas altitude is estimated from the barometer. A series of thresholds on acceleration, angular velocity, and altitude results in the detection of different fall phases. Rather than using fusion techniques to filter sensor data, Figuereido in [22] exploits the software sensors of a smartphone. In particular, features from acceleration, gyroscope, linear acceleration, and orientation sensors are compared with thresholds to allow the detection of falls. More recent evidence [23] shows that employing more sensors offers adequate amount of data to use ML. This work proves that the combination of an IMU sensing device attached to the waist with a Support Vector Machine (SVM) classification method generates better results (sensitivity = 99.50%, specificity = 99.19%).

A growing body of literature has evaluated the fusion of data from different sensors on multiple locations. The works of Li [24] and Nyan [25] are the earliest studies found in the literature that place sensor nodes on two body locations. In [24], two sensing devices comprised of accelerometer and gyroscope were attached to the chest and the thigh measuring the trunk-gravity and thigh-gravity angles. Then, thresholds differentiate between intentional and unintentional transitions, the latter indicating the incidence of falls. In [25], the correlation between the two angles (i.e., trunk-gravity, thigh-gravity) and the correlation of angular velocities with the corresponding patterns were the indicators of fall event. The contribution of Özdemir [26] is considered a breakthrough in the fall detection literature, since their analysis finds which motion sensors and which axes of these sensors contain the most relevant information for fall detection. Using PCA, the initial feature vector is reduced, while a classifier performance analysis indicates that kNN outperforms other classifiers (sensitivity = 100%, specificity = 99.79%). Another notable work is the approach of Ordóñez [27], who describes a deep learning architecture for the general problem of activity recognition. Yet, in order to exploit the ability of dense neural networks (NN) to extract activity characteristics, his procedure involves 19 sensor units (7 IMUs and 12 accelerometers) distributed on the human body. Finally, of particular mention is the work in [28], since the features extracted from the on-body IMU nodes are combined together with a Kalman filter and a control algorithm that involves user profiles that describe the individual’s motion parameters. The detection accuracy of normal falls is 99.50%, but it greatly decreases for dampened falls.

Another area of data fusion of particular interest is the combination of wearable and ambient sensors. These methods usually tackle not only the problem of fall detection but also human activity monitoring. The algorithm of [29] combines video frames from a camera with acceleration and angular velocity from an ear-worn IMU. The fusion is performed at the decision level at which a spatial Hidden Markov Model (HMM) determines the likelihood of a posture and then a temporal HMM detects if a given pattern (sequence of postures) has occurred. In addition to visual and motion data, the work of [30] incorporates sound data acquired from installed microphone arrays and on-body microphones. A set of features extracted from each sensor type is used by an abstract semantic language to construct event detection rules. The authors of [31] utilize the Dempster-Shafer’s theory to fuse the outputs of two monitoring systems. What these systems essentially do is to detect lying posture, movement, and fall-like events that constitute the nodes of a belief network that evolves in time using an algorithm based on HMMs. The analysis of the performance of the above algorithms concludes that combining more than one detection system offers a significant performance improvement compared to the single systems.

Even though there are many algorithms that perform well on a target dataset, their performance may not be good enough in another dataset. Also, only a few algorithms have been evaluated on various datasets, so direct comparisons between them cannot be made. The reason is that each study uses its own dataset created in a different way [5], so the performance metrics reported in literature cannot be used to accurately compare different algorithms unless the test dataset and the implementation code are provided. In [34], the authors compare several recent publicly available datasets and they recommend the algorithms be tested using more than one datasets.

## 3. Methods

The methods selected to compare with are recent studies that utilize smartphone accelerometers and gyroscopes to address the problem of fall detection. The criteria for choosing these algorithms were: (1) have been recently published; (2) use IMU sensors, especially accelerometers and gyroscopes, which are embedded in every smartphone; (3) can be easily implemented on smartphone devices; (4) are representative of the IMU methods. The selected algorithms were published after 2015, use only accelerometers and gyroscopes, and have been implemented on smartphone devices. The difference between them resides in how they combine the collected data. The first algorithm [13] uses only acceleration data, acceleration magnitude, and acceleration on the vertical direction (computed using angular velocity). On the other hand, the second algorithm [22], in addition to the acceleration signal, uses directly the gyroscope data to apply a threshold. The third algorithm [20] works only with the raw data in each axis, which is compared with pattern signals. Regarding the fusion technique employed, the first and the third algorithm belong to the direct data fusion level, while the second is feature level fusion method. These three methods are compared with our previous work [32], in which we consider an extension of TBA based on the use of a single accelerometer. In this way, we evaluate whether the fusion of accelerometer and gyroscope improves the fall detection performance. The implementation and comparison were carried out in MATLAB^®^, in which any parameters were chosen following the procedure described in the corresponding papers.

### 3.1. Algorithm 1 (Alg. 1)

The first work that we will describe is that of Casilari et al. [13]. This study compares four TBAs using IMU data from smartphone and smartwatch. Accelerometer and gyroscope data are collected from the smartphone placed inside the right pocket and the smartwatch. Considering the limited capabilities especially of smartwatches, these techniques employ simple thresholds. Of these methods, the proposed ‘Two-phase detection’ combines accelerometer and gyroscope data in the direct data fusion method. The decision is based on the signal magnitude vector (SMV) and the absolute value of absolute vertical acceleration (AV), given by:

(1) $$\left|\mathrm{SMV}\right|=\;{\left({\left|A_{x}\right|}^{2}\;+\;{\left|A_{y}\right|}^{2}\;+\;{\left|A_{z}\right|}^{2}\right)}^{1/2}$$

(2) $$\left|\mathrm{AV}\right|\;=\;\left|A_{x}\;\sin\theta z\;+\;A_{y}\;\sin\theta y\;-A_{z}\;\cos\theta y\;\cos\theta z\right|$$

**Figure 2:**
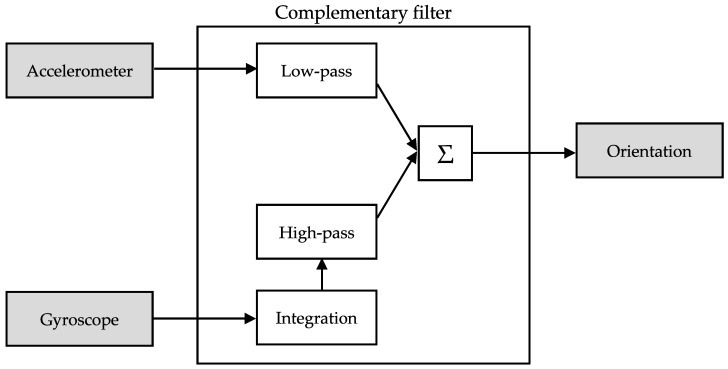
Block diagram of the complementary filter. The complementary filter removes noise (low-pass) from the accelerometer data and eliminates drift (high-pass) of gyroscope data.

The fusion is realized in the calculation of the angles θ_z_ and θ_y_, which represent the roll and pitch orientation of the smartphone. In our implementation of the algorithm, we compute these values with a complementary filter [2] (Figure 2). This type of fusion works by integrating the gyroscope data to get an angle, θ_g_, then applies a high-pass filter to the result and adds it to low-pass filtered angle estimation of the accelerometer, θ_a_. The advantage of using this type of filter to estimate the orientation is that it removes the noise from the acceleration and eliminates angular velocity drift. The fusion equations that calculate the next orientation angles θ’_z_ and θ’_y_ are given by:

(3) $${\theta ’}_{z}\;=\;\alpha \;\left(\theta_{z}\;+\;\theta_{\mathrm{gz}}\right)\;+\;\left(1\;-\;\alpha \right)\;\theta_{\mathrm{az}}\;{\theta ’}_{y}\;=\alpha \;\left(\theta_{y}\;+\;\theta_{\mathrm{gy}}\right)\;+\;\left(1\;-\;\alpha \right){\;\theta }_{\mathrm{ay}}$$

in which θ_gz_ = G_z_ Δt, θ_az_ = atan2(A_x_, sqrt(A^2^_y_ + A^2^_z_)), θ_gy_ = G_y_ Δt, θ_ay_ = atan2(−A_y_, sqrt(A^2^_x_ + A^2^_z_)).

In the above equation, the constant α controls how much of the two components are merged; when α = 1, only gyroscope data are used, whereas when α = 0, the angle is estimated solely from the accelerometer data. Typically, the value of α is between 0.9 and 0.98, since we want to favor the estimation from the less noisy gyroscope data.

The algorithm analyses the fall in two stages i.e., free-fall and impact. In the first stage, the maximum difference of SMV in a time interval of 0.1 s is compared against a threshold, and, if exceeded, the comparison is repeated within an interval of 1 s to detect the impact. This procedure is performed for the AV, as well. If both phases are detected by both SMV and AV, a fall is assumed. In addition, the algorithm processes data from a smartwatch and detects a fall only if both devices detect a fall within 1 s. However, due to lack of IMU data from smartwatch in our datasets, these additional computations are not performed.

MATLAB^®^ was used to find the threshold values for SMV and AV in each phase. This was performed with random search in each fold of cross-validation, while the Receiver Operating Characteristic (ROC) theory gives the optimum thresholds (see Section 4). Table 2 shows the search range and the optimum threshold values:

### 3.2. Algorithm 2 (Alg. 2)

In their work, Figuereido et al. [22] investigate how the hardware (accelerometer, gyroscope) and software (linear acceleration, orientation) sensors of a smartphone can help in fall detection. They use a smartphone attached inside a trouser pocket or in a belt that collects data from accelerometer, gyroscope, and magnetometer, as well as linear acceleration and orientation of the device. In their feature fusion method, Alg. 4, acceleration and angular velocity are low-pass filtered, and then a set of 5 features is extracted from a 2 s sliding window. These values are then compared with thresholds. The set of features is comprised of the sum of the absolute acceleration components (SV), the angle variation (AV), the change in orientation before and after a fall (CA), the sum of the absolute components of angular velocity (SV_G_), and the sum of the absolute components of angular acceleration (SV_GA_). Each of the above features is extracted using data from only the accelerometer or the gyroscope.

The algorithm sets a sliding window and applies a low-pass filter to acceleration and angular velocity. Then, in every sliding window, it finds the maximum value of the SV and its time index (t_SV_) and compares the former with the corresponding threshold. Next, if it surpasses the threshold it calculates the other features from a smaller time-window (for AV, SV_G_, and SV_GA_ the time-window is (t_SV_ − 1, t_SV_ + 1) s and for CA it uses before fall window (t_SV_ − 2, t_SV_ + 1) s and after fall window (t_SV_ + 1, t_SV_ + 2) s) and compares them with the corresponding thresholds. If all the values are greater than the thresholds, a fall is detected. The equations for the calculation of the features are shown below:

(4) $$\mathrm{SV}=\left|A_{x}\right|\;+\;\left|A_{y}\right|\;+\;\left|A_{z}\right|$$

(5) $$\mathrm{AV}\;=\;\left[\cos^{-1}\left(A^{n}\;A^{n+1}\;/\;\|A^{n}\|\;\|A^{n+1}\|\right)\right]\;180/\pi ,\;\mathrm{for}\;n\;=\;1,\ldots ,N-1$$

(6) $$\mathrm{CA}\;=\;\left[\cos^{-1}\left({Ā}_{b}\;{Ā}_{e}\;/\;\|{Ā}_{b}\|\;\|{Ā}_{e}\|\right)\right]\;180/\pi$$

(7) $${\mathrm{SV}}_{G}=\;\left|G_{x}\right|+\;\left|G_{y}\right|\;+\;\left|G_{z}\right|$$

(8) $${\mathrm{SV}}_{\mathrm{GA}}\;=\;\left|{\mathrm{GA}}_{x}\right|\;+\;\left|{\mathrm{GA}}_{y}\right|\;+\;\left|{\mathrm{GA}}_{z}\right|$$

where A = (A_x_, A_y_, A_z_) is the acceleration vector, the symbol ‖‖ is the Euclidean norm, N is the number of samples in each 2 s time-window, Ā_b_, Ā_e_ are the average acceleration vectors before and after the fall, G = (G_x_, G_y_, G_z_) is the angular velocity vector, and GA = (GA_x_, GA_y_, GA_z_) is the vector of angular acceleration approximated by the forward differences GA^n^ = (G^n+1^ − G^n^)/Δt for n = 1,…, N − 1.

In our implementation, we chose the thresholds for each feature according to ROC theory using a random search in each fold (see Section 4). The search range and the optimum threshold values are reported in Table 3.

### 3.3. Algorithm 3 (Alg. 3)

The third method we will present is the study of Ando et al. [20]. In their work, they try to perform activity recognition between 9 activities using accelerometer and gyroscope data from a smartphone worn at the hip. The proposed direct data fusion technique is more complicated than the others considering that it is based on thresholding the correlation and it requires some preprocessing. The latter aims to calculate a 5 s pattern for each activity, which is then correlated with a signal segment.

The algorithm starts by checking whether the correlation of a data segment with an activity pattern exceeds a threshold (TH_CA_). This is repeated for all available patterns, creating a vector R^Acc^ of size equal to the number of activities and values 1 for exceeding the threshold and 0 otherwise. If there are more than one activity matches—that is, cases in which the correlation with other patterns exceeds the threshold—gyroscope data are used to refine the classification. Firstly, the angular velocity G_z_ is compared against another threshold (TH_GZ_), and, if it is meaningful, the correlation between the angular velocity on the z-axis and the corresponding pattern of all the activities is thresholded (TH_CGZ_), creating a vector R^Gyr,z^ similar to R^Acc^. Then, the vector R = R^Acc^ & R^Gyr,z^ is used for the classification. In case the G_z_ component is meaningless, the same procedure is applied to G_Y_.

We have replicated this algorithm in MATLAB^®^ using only an activity pattern—that of falls. Additionally, in case of a match with the accelerometer data, the gyroscope patterns are correlated with the measured angular velocity. In our approach, we consider the thresholds of the angular velocities to be equal in between the two axes (TH_GZ_ = TH_GY_ = TH_G_ and TH_CGZ_ = TH_CGY_ = TH_CG_). The threshold values were chosen according to ROC theory (see Section 4) and are reported in Table 4.

### 3.4. Algorithm 4 (Alg. 4)

In our previous research [32], we studied the problem of fall detection using only accelerometer data collected from a smartphone. This algorithm uses a threshold-based method to detect fall-like segments, and then from these segments, a set of features is extracted and forwarded to a kNN classifier to refine the classification.

**Figure 3:**
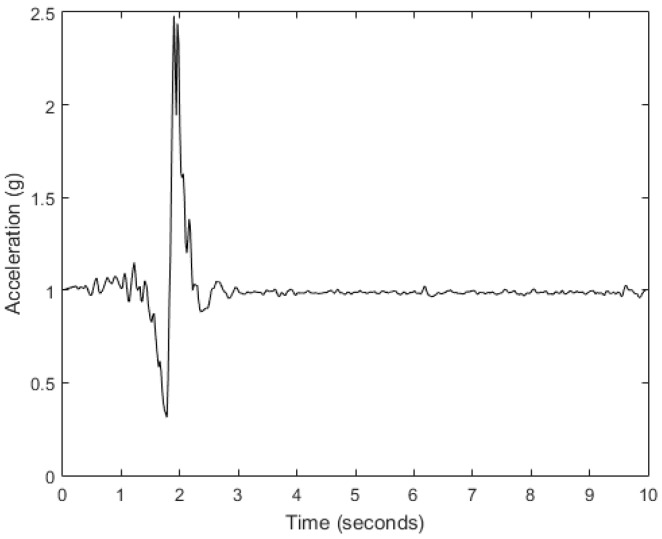
Typical waveform of fall event from accelerometer sensor.

This method is based on the detection of possible falls performed by incorporating both acceleration magnitude thresholds and timers in a finite state machine (FSM) to model the fall phases of the typical waveform (Figure 3) of a fall event described in [35]. If a possible fall is found, a set of features is created from a segment around the impact indicated by the maximum of the acceleration. The features try to cover many characteristics of the fall pattern from the time and frequency domains while a kNN makes the final decision. The parameters most important to the detection algorithm are the peak value of the acceleration magnitude (SMV_PEAK_); the time interval of inactivity detection ([AT_LOW_,AT_HIGH_]) after the acceleration peak; the feature extraction segment ([FS_LOW_,FS_HIGH_]), which should include data around the peak; and the number of nearest neighbors of the classifier (k).

The algorithm was implemented in MATLAB^®^ using only the acceleration signals of each dataset. The threshold values were chosen based on ROC theory (see Section 4) and are reported in Table 5.

## 4. Experiments

The performance of the above-mentioned algorithms was assessed with a systematic analysis. We used accelerometer and gyroscope data from three publicly available datasets: MobiAct, DLR, and UMAFall. The first version of the MobiAct dataset [36] consists of 4 types of falls (forward, forward with first impact on knees, sideward, backward) and 9 types of ADLs (standing, walking, jogging, jumping, stairs up/down, sit, car step in/out) collected at 100 Hz sampling rate by a smartphone loosely attached in a trousers pocket. Falls were simulated on a 5 cm mattress while the subjects carried the phone in the pocket on the opposite side of falling. The DLR dataset [37] contains 6 types of ADLs (running, walking, jumping, standing, sitting, and lying) and 1 arbitrary fall. The sensor node is located on a belt around the waist and collects data at 100 Hz. The UMAFall dataset [38] consists of 8 different types of ADLs (squatting, climbing stairs down/up, hopping, jogging, lying on bed, getting up from bed, sitting on a chair, getting up from a chair, walking) and 3 types of falls (forward, backward, lateral). It uses 4 sensor nodes and a smartphone to collect data. The sensor nodes consist of accelerometer, gyroscope, and magnetometer placed at the chest, the waist, the wrist, and the ankle, and use a sampling rate of 20 Hz. The smartphone is placed inside the right pocket and collects acceleration, angular velocity, and magnetic field data at 200 Hz.

The accelerometer and gyroscope values were downsampled at 50 Hz and preprocessed with a median low-pass filter of 3 samples to smooth the data. We chose this sampling frequency as a compromise between high fidelity in our measurements and low consumption. According to literature, the spectrum range of human is between 0 Hz and 20 Hz [9,39], so at least 40 Hz sampling is required to satisfy the Nyquist-Shannon theorem. On the other hand, the hardware of most smartphones limits the highest sampling rate to 100 Hz. Therefore, considering that in a real-world application for smartphones the battery drain should be taken into account and that higher sampling rate results in increased power consumption, we preferred sampling at 50 Hz, which is also used in other similar works.

**Figure 4:**
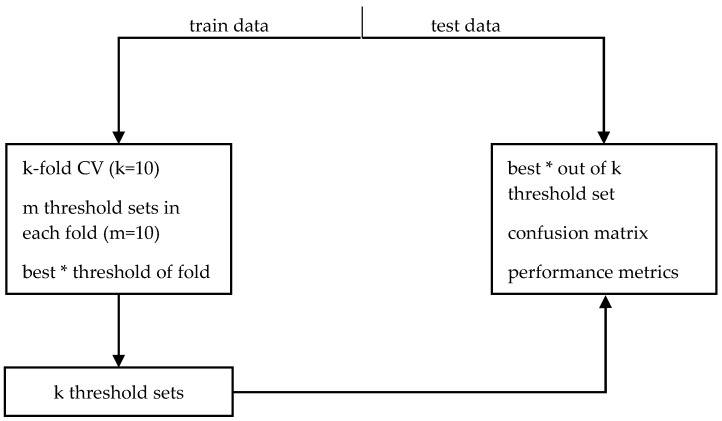
Block diagram of threshold selection procedure. (*) The best thresholds are the ones that yield a performance close to (0, 1) in the ROC curve.

**Figure 5:**
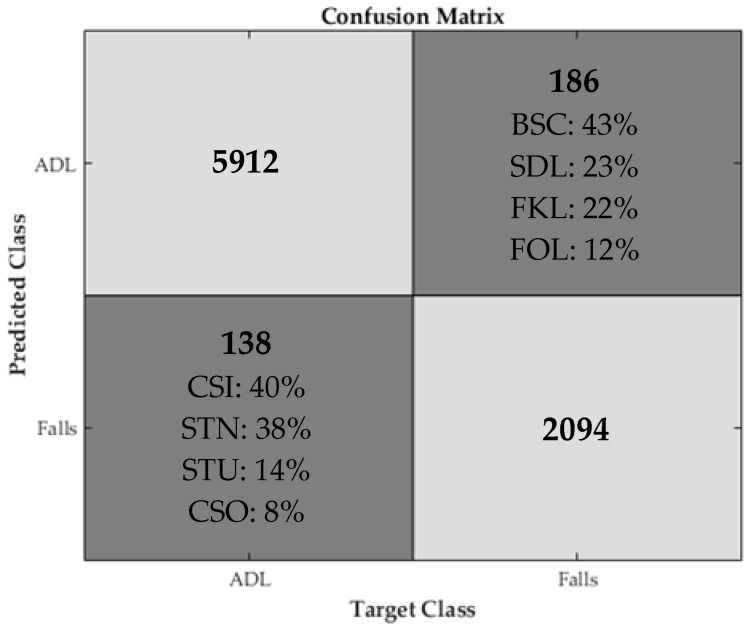
Cumulative confusion matrix of Alg. 4 with data from the MobiAct dataset. For the wrong predictions the percentage of each ADL (CSI: Step-into car, CSO: Step-out car, STN: Go downstairs, STU: Go upstairs) and fall (BSC: Back-sitting-chair; FKL: Front-knees-lying; FOL: Forward-lying; SDL: Sideward-lying) type is reported.

The appropriate threshold values required by the algorithms were found by a procedure based on cross-validation (Figure 4). To that purpose, for every algorithm and dataset, we repeated the following procedure. The sensor data of a dataset are split into a train and a test set. The train set is used to choose the best thresholds, that is, the thresholds that maximize the detection performance. In every fold of a 10-fold Cross Validation (CV), we randomly choose 10 sets of threshold values and select the best set of thresholds. Then, the best threshold set was determined by means of ROC theory between the 10 sets that have been selected from all folds. This means the threshold set that resulted in a point closer to (0, 1) on the Sensitivity vs. (1-Specificity) plot was reported (Table 2, Table 3, Table 4 and Table 5) as the best. The threshold sets from all folds along with the test set are used to create the confusion matrix, i.e., for each pair of algorithm and dataset we created 10 confusion matrices using the best thresholds of each fold that were used to compute the performance metrics described in the next section. Figure 5 shows the cumulative confusion matrix of Alg. 4 with data from the MobiAct dataset.

All the code development required for the experiments, as well as for the statistical analysis described in the next section, were performed in MATLAB^®^.

## 5. Results and Discussion

The aforementioned algorithms are compared across three datasets: MobiAct, DLR, and UMAFall. The MobiAct dataset [36] contains acceleration and angular velocity data for 4 types of falls and 9 types of ADLs sampled at 100 Hz sampling rate by a smartphone loosely attached in a trousers pocket. The DLR dataset [37] is comprised of 6 types of ADLs and 1 type of arbitrary fall. Data are collected by a sensor node located on a belt around the waist at a rate of 100 Hz. The UMAFall dataset [38] consists of 8 different types of ADLs and 3 types of falls. The sensor nodes as well as a smartphone measure acceleration, angular velocity, and magnetic field at 20 Hz and 200 Hz respectively.

For our analysis, we have used the data collected from smartphone inside a pocket where available (for MobiAct) or data from nodes attached on the belt (for DLR and UMAFall). To evaluate the performance of the above methods the following sensitivity and specificity metrics have been used:

(9) Sensitivity = Recall = TP/(TP + FN)

(10) Specificity = TN/(TN + FP)

(11) Precision = TP/(TP + FP)

(12) F1 = 2 × (Precision × Recall)/(Precision + Recall)

in which TP, TN, FP, and FN correspond to true positives, true negatives, false positives, and false negatives values obtained from the confusion matrix. A 10-fold cross-validation was performed for each dataset, the results of which are presented in Table 6, Table 7 and Table 8.

As we can see, the methods differ in detection accuracy. Of the three TBAs, Alg. 2 shows the highest performance regarding all metrics across datasets. The reason for this is the fact that Alg. 2 uses more features to detect the fall pattern characteristics compared to Alg. 1. Additionally, Alg. 3 requires the sensors to be mounted on the body in order to measure accurately the angular velocity in each axis. Thus, the performance of this threshold technique deteriorates for the loose pant pocket setting in MobiAct and UMAFall datasets, as stated in [35]. Furthermore, the impact of gyroscope drift on the detection was not taken into account in any of these methods.

Apart from differences in fusion techniques, the performance of the algorithms varies due to differences in sensor positions in the datasets and the algorithm implementation. Table 9 shows any mismatches that appear regarding the sensor position. For example, Alg. 4 assumes data from sensor inside a trousers pocket that matches the MobiAct dataset. On the other hand, the sensor position of the DLR and UMAFall datasets matches only the position in Alg. 3. These comments partly explain why the performance of Alg. 2 is lower (with a reduced mean value and higher variance) when tested on the DLR dataset. However, Alg. 3 does not show any significant improvement on the DLR dataset as it would be expected. In addition, differences arise from the fact that the datasets do not contain the same type of ADLs and falls. For instance, the DLR consists of 1 arbitrary fall, while both the other two datasets contain forward, backward, and lateral falls.

**Figure 6:**
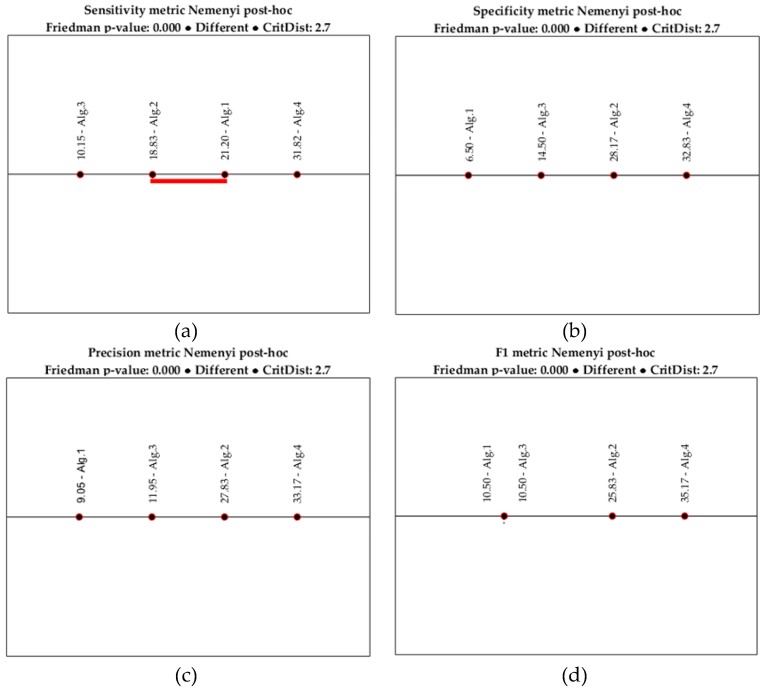
Nemenyi post-hoc tests for the performance metrics: (**a**) sensitivity; (**b**) specificity; (**c**) precision; and (**d**) F1. The red horizontal line in the sensitivity metric test denotes that the Alg. 1 and Alg. 4 are not different.

To examine whether the differences in performance between the algorithms is significant, we performed a statistical test. Particularly, the Friedman test, a non-parametric counterpart of ANOVA, followed by the Nemenyi post-hoc test, were used. The former compares the average ranks of algorithms across the datasets, while the latter is used to perform pair-wise comparisons when the null hypothesis (i.e., that there is not any significant difference between the algorithms) is rejected [40]. We applied this statistical procedure to every performance metric separately. The results are shown in Table 10, Table 11, Table 12 and Table 13 and Figure 6.

As we can see, the *p*-value is lower than the significance level α = 0.05 for every metric. So, the null hypothesis is rejected, meaning that at least two algorithms are significantly different. Performing the Nemenyi test for each case (Figure 6) shows that there is a notable difference in all pairwise comparisons. However, for the sensitivity metric, the difference between Alg. 1 and Alg. 2 is not important, since the difference between the corresponding average ranks is less than the critical distance, as is denoted by the horizontal line that connects the two algorithms (Figure 6a). The same holds for the F1 metric and algorithms Alg. 1 and Alg. 3 (Figure 6d).

Considering the three TBAs, the sensitivity measure of Alg. 1 is very close to that of Alg. 2. However, the distance of Alg. 2 from the rest is longer than the distance between Alg. 1 and Alg. 3 for the specificity, precision, and F1 metrics. This confirms that fusing data in a higher level contributes positively to the performance of the classification algorithm. Compared to our previous work [32], none of the three threshold-based methods produce satisfactory results considering the calculated performance measures. However, the distance from Alg. 2 for the specificity metric is less than two times the critical distance. Nevertheless, it is evident that an ML algorithm can be more accurate than a complex TBA, since the latter may require many parameters to be considered and adjusting all the threshold values is not always feasible.

## 6. Conclusions

In conclusion, fall detection systems constitute an important solution to the ageing population problem. Falls among the elderly can be severe enough to lead the senior to a debilitating condition. In this paper, we briefly present the main points of data fusion studies related to fall detection. Additionally, a comparison of three data fusion algorithms with a simple ML classifier is performed. The results show that the fusion methods differ in their performance, which is affected by the employed dataset, while ML techniques can improve the detection capability. Hence, the presented analysis and comparison of these methods provide useful insights into the problem of fall detection.

## Figures and Tables

**Table 1:** Literature review of data fusion in fall detection.

Ref.	Method	Sensors	Performance Sensitivity, Specificity
[7]	ML	accelerometer, gyroscope, PIR	0.7 (accuracy)
[8]	ML, TBA	accelerometer, Kinect	0.99, 1.00
[9]	TBA	accelerometer	1.00, 1.00
[10]	TBA	accelerometer	0.97, 1.00
[11]	TBA	accelerometer	0.97, 0.91
[12]	ML	accelerometer	-
[13]	TBA	accelerometer, gyroscope	0.93, 0.90
[14]	TBA	accelerometer, gyroscope	0.95, 0.94
[15]	TBA	accelerometer, gyroscope	-
[16]	ML	accelerometer, gyroscope	0.95, 0.97
[17]	TBA	accelerometer, gyroscope	0.96, 0.96
[18]	ML	accelerometer, gyroscope	0.94, 0.99
[19]	TBA	accelerometer, gyroscope	-
[20]	TBA	accelerometer, gyroscope	0.78, 0.99
[21]	TBA	accelerometer, gyroscope, barometer, magnetometer	1.00, 0.99
[22]	TBA	accelerometer, gyroscope	1.00, 0.94
[23]	ML	accelerometer, gyroscope, magnetometer	0.99, 0.98
[24]	TBA	accelerometer, gyroscope	0.91, 0.92
[25]	TBA	accelerometer, gyroscope	0.95, 1.00
[26]	ML	accelerometer, gyroscope, magnetometer	1.00, 1.00
[27]	ML	accelerometer, gyroscope, magnetometer	-
[28]	ML	accelerometer, gyroscope, magnetometer	0.99 (accuracy)
[29]	ML	accelerometer, gyroscope, camera	0.75 (accuracy)
[30]	ML	accelerometer, camera, microphone	0.96, 0.91
[31]	ML	accelerometer, IR, ECG	0.97, 0.80
[32]	TBA, ML	accelerometer	0.96, 0.97

**Table 2:** Threshold values of Alg. 1 for the MobiAct dataset.

	SMV_FF_ (g)	SMV_IP_ (g)	AV_FF_ (g)	AV_IP_ (g)
Search range	(0.7, 0.9)	(1.5, 1.7)	(0.3, 0.7]	(0.7, 1.1)
Optimum	0.8362	1.5731	0.5633	0.7387

**Table 3:** Threshold ranges and values of Alg. 2 for the MobiAct dataset.

	SV (g)	AV (°)	CA (°)	SV_G_ (rad/s)	SV_GA_ (rad/s^2^)
Search Range	(1.7, 2.3)	(21, 23)	(42, 46)	(2, 2.4)	(36, 40)
Optimum	2.0422	21.4522	42.6328	2.3986	39.6284

**Table 4:** Threshold ranges and values of Alg. 3 for the MobiAct dataset.

	TH_CA_	TH_G_ (rad/s)	TH_CG_
Search range	(0.4, 0.8)	(0.3, 0.7)	(0.2, 0.6)
Optimum	0.7684	0.3331	0.3216

**Table 5:** Threshold ranges and values of Alg. 4 for the MobiAct dataset.

	SMV_PEAK_ (g)	AT_LOW_ (ms)	AT_HIGH_ (ms)	FS_LOW_ (ms)	FS_HIGH_ (ms)	k
Search range	(1.8, 2.2)	(1000, 2000)	(2500, 3000)	(−2500, −1500)	(500, 1500)	(1, 9)
Optimum	1.8618	1423	2702	−2170	1155	7

**Table 6:** A 10-fold cross-validation of the presented algorithms using the MobiAct dataset. The table shows the average values and the standard deviations in parentheses.

	Alg. 1	Alg. 2	Alg. 3	Alg. 4
Sensitivity	0.7509 (0.0168)	0.9895 (0.0031)	0.7066 (0.0761)	0.9184 (0.0242)
Specificity	0.6856 (0.0106)	0.9283 (0.0076)	0.7760 (0.1127)	0.9772 (0.0108)
Precision	0.4738 (0.0040)	0.8389 (0.0140)	0.5650 (0.1005)	0.9388 (0.0277)
F1	0.5810 (0.0059)	0.9080 (0.0083)	0.6279 (0.0690)	0.9285 (0.0184)

**Table 7:** A 10-fold cross-validation of the presented algorithms using the DLR dataset. The table shows the average values and the standard deviations in parentheses.

	Alg. 1	Alg. 2	Alg. 3	Alg. 4
Sensitivity	0.8750 (0.0000)	0.6250 (0.0000)	0.7625 (0.0395)	0.9875 (0.0395)
Specificity	0.8230 (0.0054)	0.9827 (0.0043)	0.8775 (0.0229)	1.0000 (0.0000)
Precision	0.1717 (0.0042)	0.6075 (0.0579)	0.2112 (0.0348)	1.0000 (0.0000)
F1	0.2870 (0.0059)	0.6161 (0.0298)	0.3308 (0.0428)	0.9937 (0.0200)

**Table 8:** A 10-fold cross-validation of the presented algorithms using the UMAFall dataset. The table shows the average values and the standard deviations in parentheses.

	Alg. 1	Alg. 2	Alg. 3	Alg. 4
Sensitivity	0.9516 (0.0000)	0.9355 (0.0000)	0.5661 (0.0193)	0.9790 (0.0109)
Specificity	0.6700 (0.0045)	0.9714 (0.0000)	0.7900 (0.0399)	0.9586 (0.0157)
Precision	0.7186 (0.0027)	0.9667 (0.0000)	0.7069 (0.0364)	0.9547 (0.0163)
F1	0.8189 (0.0018)	0.9508 (0.0000)	0.6287 (0.0187)	0.9667 (0.0099)

**Table 9:** Sensor type position in datasets/algorithms. Greyed cells represent a matching sensor position between the dataset and the algorithm.

	Alg. 1	Alg. 2	Alg. 3	Alg. 4
MobiAct	pocket/pocket (and wrist)	pocket/pocket (or belt)	pocket/belt	pocket/pocket
DLR	belt/pocket (and wrist)	belt/pocket (or belt)	belt/belt	belt/pocket
UMAFall	belt/pocket (and wrist)	belt/pocket (or belt)	belt/belt	belt/pocket

**Table 10:** Friedman test table for the sensitivity metric.

Source	SS	df	MS	Chi-sq	Prob > Chi-sq
Columns	7153.72	3	2384.57	54.43	9.0961 × 10^−12^
Interaction	7088.53	6	1181.42		
Error	1135.75	108	10.52		
Total	15,378.00	119			

**Table 11:** Friedman test table for the specificity metric.

Source	SS	df	MS	Chi-sq	Prob > Chi-sq
Columns	13,286.70	3	4428.89	99.29	2.2084 × 10^−21^
Interaction	973.30	6	162.22		
Error	1396.50	108	12.93		
Total	15,656.50	119			

**Table 12:** Friedman test table for the precision metric.

Source	SS	df	MS	Chi-sq	Prob > Chi-sq
Columns	12,552.80	3	4184.27	93.39	4.0857 × 10^−20^
Interaction	1016.40	6	169.41		
Error	2156.30	108	19.97		
Total	15725.50	119			

**Table 13:** Friedman test table for the F1 metric.

Source	SS	df	MS	Chi-sq	Prob > Chi-sq
Columns	13,306.70	3	4435.56	98.86	2.7342 × 10^−21^
Interaction	853.30	6	142.22		
Error	1588.50	108	14.71		
Total	15,748.50	119			

**SS**: sum of squares; **df**: degrees of freedom; **MS**: mean squares (SS/df); **Chi-sq**: chi-square statistic value; **Prob > Chi-sq**: *p*-value.
